# Associations Between Sleep Quality and Self-Reported Health Status in Middle-Aged and Older Adults: A Community-Based, Cross-Sectional Study in Northern Taiwan

**DOI:** 10.3390/healthcare13111272

**Published:** 2025-05-28

**Authors:** Wen-Hsueh Chen, Chao-Tung Chen, Kai-Hung Cheng, Yu-Chung Tsao, Yu-Hsiang Lin, Jau-Yuan Chen

**Affiliations:** 1 Department of Family Medicine, Chang-Gung Memorial Hospital, Chiayi Branch, Chiayi 613, Taiwan; dr8732@cgmh.org.tw (W.-H.C.); peterc@cgmh.org.tw (C.-T.C.); 2 Department of Family Medicine, Chang-Gung Memorial Hospital, Linkou Branch, Taoyuan 333, Taiwan; b100034@cgmh.org.tw; 3 Department of Occupational Medicine, Chang-Gung Memorial Hospital, Linkou Branch, Taoyuan 333, Taiwan; eusden@cgmh.org.tw; 4 College of Medicine, Chang-Gung University, Taoyuan 333, Taiwan; laserep@cgu.edu.tw; 5 Master of Science Degree Program in Innovation for Smart Medicine, Chang-Gung University, Taoyuan 333, Taiwan; 6 College of Life Sciences and Medicine, National Tsing Hua University, Hsinchu 300, Taiwan; 7 Department of Urology, Chang-Gung Memorial Hospital, Linkou Branch, Taoyuan 333, Taiwan; 8 Department of Health Management and General Practice, Xiamen Chang-Gung Hospital, Xiamen 361028, China

**Keywords:** sleep quality, self-reported health status, community-based, middle-aged and older people

## Abstract

**Background/Objectives**: Poor sleep quality is a prevalent health concern among older adults, impacting cognitive and physical functions. This study aimed to determine the association between sleep quality and self-reported health status among middle-aged and older adults in northern Taiwan. **Methods**: This cross-sectional study, conducted from April to October 2017, assessed participants using the Chinese version of the Pittsburgh Sleep Quality Index (CPSQI) with a cut-off of 5; scores above 5 indicated poor sleep quality. The self-reported health status was evaluated using a questionnaire. Statistical analyses included the chi-squared test, one-way ANOVA, Cochran–Armitage trend test, and multiple logistic regression models. **Results**: This study included 850 adults (243 males and 607 females). The participants were grouped according to their self-reported health status as follows: good (n = 278), fair (n = 499), and poor (n = 73). Poor health status was associated with worse sleep quality components, including sleep latency, efficiency, disturbances, medication use, and daytime dysfunction (*p* for trend < 0.001). The multiple logistic regression analysis showed higher dissatisfaction with health status among the participants with a CPSQI score of >5 (odds ratio, 4.12; 95% CI 2.26–7.50; *p* < 0.001). A poor health status was reported by 19.51% of the participants sleeping < 5 h, compared to 6.97% of the participants sleeping 5–6 h, 6.60% of the participants sleeping 6–7 h, and 6.34% of the participants sleeping > 7 h, showing a trend toward a shorter sleep duration (*p* for trend = 0.002). **Conclusions**: Our study findings indicate that a poor sleep quality and short sleep duration were independent risk factors for poor self-reported health status in middle-aged and older adults in Taiwan. Addressing sleep quality is crucial for implementing preventive health measures in this demographic group.

## 1. Introduction

Sleeping problems have become a significant global public health issue, with studies showing that the prevalence of sleeping problems is 56% in the United States, 31% in Western Europe, and 23% in Japan [1]. In Taiwan, poor sleep quality affects 46.6% of the population [2]. Sleep disorders are associated with multiple aspects of health outcomes. Itani et al. performed a meta-analysis and reported that short sleep durations were significantly associated with several chronic diseases, such as diabetes mellitus (DM), hypertension (HTN), and obesity [3]. Krittanawong et al. and Yin et al. revealed an association between sleep duration and cardiovascular disease (CVD), with implications for all-cause mortality [4,5]. Kwok et al. also reported that the self-reported sleep quality significantly correlated with CVD incidence [6]. Longitudinal evidence has suggested a bidirectional association between sleep disturbances and urological symptoms, such as nocturia and incontinence [7]. Short sleep durations increase the risk of upper respiratory tract infections [8] and have been associated with a higher incidence of nonalcoholic fatty liver disease (NAFLD) in cohort studies [9]. Individuals with insomnia show small-to-moderate impairments in executive functions, including working memory and cognitive flexibility [10]. In addition, a recent meta-analysis found that the self-reported sleep quality was positively associated with the quality of life among older adults [11].

The self-reported health status is a widely used, reliable, and simple indicator of general health. It reflects an individual’s perception of physical, mental, and social well-being and is predictive of future morbidity and mortality [12,13]. Several studies have investigated the association between sleep characteristics and the self-rated health status. Li et al. examined Chinese university students and found that short sleep durations (<6 h) were independently associated with poor self-rated health after adjusting for psychosocial and academic variables [14]. Štefan et al. demonstrated that both short (<7 h) and long (>10 h) sleep durations were associated with lower odds of good self-rated health among young adults [15]. Andreasson et al. reported that a long sleep duration was associated with worse self-rated health among individuals who reported poor sleep quality [16]. A recent meta-analysis of 26 studies confirmed that both short (≤8 h) and long (>8 h) sleep durations were significantly associated with worse self-rated health [17].

However, current research on sleep and self-rated health still presents several gaps. First, the findings remain inconsistent. Some studies have linked poor health to short sleep durations, while others have linked it to long sleep durations. Second, much of the literature is based on Western populations, and translating these results to Asian populations, specifically Taiwanese populations, may not be entirely appropriate because of potential differences in cultural, social, and lifestyle factors. Third, studies examining this association in older populations are limited. Therefore, this study aimed to investigate the correlation between sleep quality and self-reported health status among community-dwelling middle-aged and older individuals in Taiwan. We hope that our findings will provide valuable insights into improving the health status of this population by improving sleep quality.

## 2. Materials and Methods

### 2.1. Study Design and Participants

This study was a community-centered, cross-sectional study conducted over a period spanning from April to October 2017. Initially, the study involved recruiting 1308 individuals, with selection based on specific inclusion criteria: (1) aged 50–85 years and (2) living in the same district for at least 6 months. Participants were excluded if they (1) lacked the ability to communicate effectively for interview purposes, (2) had recently been diagnosed with CVD, or (3) resided in a long-term care facility at the time of the study. Ultimately, the study analyzed data from 850 participants, comprising 243 males and 607 females. This study received ethical approval and validation from the Chang-gung Medical Foundation Institutional Review Board, and all participants provided signed informed consent before enrollment.

### 2.2. Data Collection

The collected data included body mass index (BMI), marital status, educational level, income, Center for Epidemiologic Studies Depression Scale (CES-D) score, employment status, exercise status, smoking status, alcohol consumption, and medical history. The BMI was calculated as the ratio of weight to height in meters squared (kg/m^2^). Depressive symptoms were measured using a previously validated shorter version of the CES-D with 10 items, where each item score ranged from 0 to 3, and the cut-off point for depressive symptoms was a score of ≥8. The personal information of the participants was gathered via face-to-face questionnaires administered by a trained research assistant. The questionnaire included questions regarding exercise habits, marital status, educational level, income, and medical history. Marital status was categorized into two distinct groups: currently single individuals, encompassing those who were divorced, separated, widowed, or never married and those currently married. Educational levels were divided into four groups: Group 1 (uneducated), Group 2 (primary school graduates), Group 3 (secondary school graduates), and Group 4 (college graduates). Participants’ exercise habits were characterized based on how frequently they exercised, with a specific focus on those who engaged in physical activity at least three times weekly for durations exceeding 30 min. The monthly income of the participants was categorized into three tiers: Group 1, earning < TWD 20,000; Group 2, with incomes ranging from TWD 20,000 to 40,000; and Group 3, earning > TWD 40,000. The past medical history included DM, HTN, or hyperlipidemia.

### 2.3. Assessment of Sleep Quality

The Pittsburgh Sleep Quality Index (PSQI), a self-reporting tool, was used to evaluate sleep quality in participants. This questionnaire comprises 19 questions divided into seven components: subjective sleep quality, sleep latency, sleep duration, sleep efficiency, sleep disturbances, use of sleep medication, and daytime dysfunction. Each component was rated on a scale of 0–3, and the aggregate of these scores yielded a comprehensive score that varied between 0 and 21. Higher scores indicated worse sleep quality. The Chinese version of the PSQI (CPSQI) is a reliable and validated tool for evaluating poor sleep quality in community-based studies, with Cronbach’s α ranging from 0.82 to 0.83. A cutoff point of 5 was used to indicate poor sleep quality and high sensitivity for primary insomnia compared with controls [18]. In this study, participants completed the CPSQI, and a CPSQI score > 5 indicated poor sleep quality. In a study by Ferrie et al., sleep duration was categorized as ≤5, 6, 7, 8, and ≥9 h based on weekday averages in a general adult population [19]. However, given that our participants were older adults, we adopted the recommendation of Hirshkowitz et al., which considers ≥ 7 h as the optimal sleep duration for older individuals [20]. Therefore, we grouped sleep duration as >7, 6–7, 5–6, and <5 h to reflect both the distribution in our sample and the age-appropriate sleep health guidelines.

### 2.4. Assessment of Self-Reported Health Status

Self-reported health status is a subjective measure of health status that combines an individual’s biological, psychological, social, and functional dimensions and is often used in epidemiological research [15,21,22]. In this study, self-reported health status was assessed using the following question: ‘In general, would you say that your health is excellent, good, fair, poor, or very poor?’. In our study, we categorized the responses into three distinct outcomes: “good” (encompassing both “excellent” and “good” answers), “fair” (solely based on the “fair” response), and “poor” (comprising “poor” or “very poor” answers) to facilitate more streamlined comparisons in the analysis. This approach has been documented in previous studies [23,24,25].

### 2.5. Statistical Analyses

One-way analysis of variance (ANOVA) was used to calculate *p*-values for continuous variables, whereas the chi-squared test was used to determine *p*-values for categorical variables. The Cochran–Armitage trend test was used to investigate trends in self-reported health status and different components of sleep quality. Multiple logistic regression models were used to explore the relationship between sleep quality and self-reported health status. All statistical calculations were conducted using IBM Statistical Product and Service Solutions Statistics for Windows (version 22.0; IBM Corp., Armonk, NY, USA). A *p*-value of <0.05 was considered to indicate statistical significance, which was corrected using the false discovery rate.

## 3. Results

Our study included 850 participants aged 50–85 years, of whom 28.59% were male and 71.41% were female. The participants were divided into three groups according to their self-reported health status: good (n = 278), fair (n = 499), or poor (n = 73). The educational levels, CES-D scores, employment status, exercise, and HTN, DM, and hyperlipidemia status differed significantly between the groups. The CES-D score reflects the severity of depressive symptoms, with higher scores indicating more severe symptoms. No significant differences were observed among the three groups in terms of the age, BMI, gender, marital status, income, smoking status, or alcohol consumption, as shown in Table 1.

Based on their general characteristics, the members of the study population were categorized into three groups—good, fair, and poor—reflecting their self-reported health status (Table 2). The self-reported health status was directly related to the subjective sleep quality (*p* for trend <  0.001). Furthermore, the self-reported health status was significantly associated with multiple sleep components, including sleep quality, latency, efficiency, disturbances, medication use, and daytime dysfunction (all *p* for trend <  0.001).

Table 3 shows the results of the multiple logistic regression analysis. Model 1 was adjusted for age and gender. Model 2 was adjusted for factors from Model 1, plus marital status, educational level, exercise status, smoking status, and alcohol consumption. Model 3 was adjusted for factors from Model 2, plus HTN, DM, and hyperlipidemia. Model 4 was adjusted for factors from Model 3, plus income, CES-D score, and employment. After adjusting for the aforementioned confounding factors, poor health status continued to be independently associated with poor subjective sleep quality (CPSQI score > 5). The adjusted odds ratio (OR) was 2.31 (95% confidence interval [CI] = 1.20–4.45). This independent association was also detected for various components of sleep, including sleep quality (OR = 1.63, 95% CI = 1.15–2.32), sleep disturbance (OR = 2.63, 95% CI = 1.51–4.56), and daytime dysfunction (OR = 1.75, 95% CI = 1.30–2.37).

**Figure 1:**
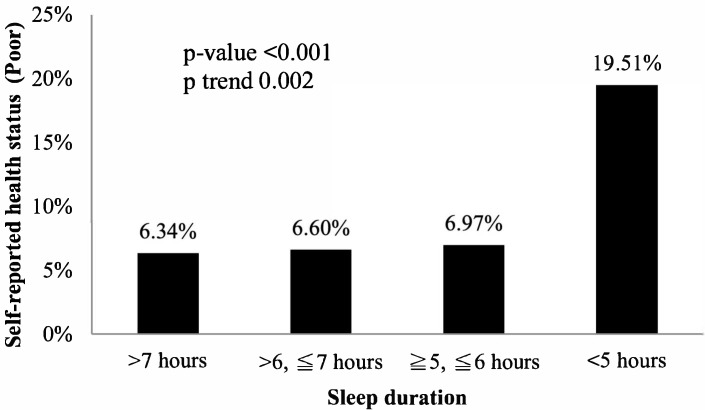
Association between sleep duration and self-reported health status.

An examination of sleep duration revealed that 19.51% of the participants who had been sleeping for <5 h reported poor health. In contrast, 6.97% of the individuals reporting 5–6 h of sleep, 6.60% of the individuals reporting >6 but up to 7 h of sleep, and 6.34% of the individuals reporting >7 h of sleep reported poor health. The data indicated a trend toward growing dissatisfaction with health status as sleep duration decreased (*p* for trend = 0.002), as depicted in Figure 1.

## 4. Discussion

Our research objective was to explore the relationship between sleep quality and self-reported health status. According to the results, poor sleep quality and short sleep duration were independent risk factors for poor self-reported health status among middle-aged and older Taiwanese individuals. These findings indicate that sleep quality is important when devising strategies for improving the health of middle-aged and older adults.

In our study, poor sleep quality was an independent factor affecting self-reported health status even after adjusting for marital status, education level, exercise, smoking, alcohol consumption, income, depression, and medical history. A previous meta-analysis revealed that poor sleep quality was associated with worse self-rated health status [16], although the mean age of the participants in that study was lower (28 years), and the studies predominantly involved Western populations. Poor sleep quality is related to a range of chronic illnesses, such as HTN, DM, CVD, obesity, and upper respiratory tract infections [3,6,8,26]. These unfavorable health conditions may further influence the self-rated health status. However, in a study involving relatively healthy participants [27], poor sleep quality negatively impacted the self-reported health. A recent cross-sectional, population-based study conducted in Brazil with 1762 adults from October to December 2020 similarly reported that individuals with poor sleep quality were 2.82 times more likely to self-assess their health as poor [28]. These findings suggest that poor sleep quality is an independent risk factor for poor self-reported health. The results of our study further support this hypothesis. In addition, a study involving Taiwanese hospital physicians found that sleep quality was influenced by gender-specific lifestyle and occupational factors; poor dietary and exercise habits were significantly associated with worse sleep quality in female physicians, whereas high job demands were a key factor for male physicians [29]. This suggests that sleep quality is shaped by a range of social and behavioral determinants, which may vary across populations and demographic subgroups and should be considered when evaluating its impact on perceived health.

Our study showed that as sleep duration decreased, the proportion of participants with poor self-reported health status increased. This finding diverges somewhat from the findings of a cross-sectional study that reported a U-shaped relationship between sleep duration and self-reported health status [30]. This discrepancy may have arisen from variations in sleep duration. In our study, sleep duration was grouped into four categories: >7, 6–7, 5–6, and <5 h. Other studies have shown that the inflection point of the U-shaped curve typically occurs at approximately 7–8 h of sleep [15,31,32]. One cross-sectional study revealed that the self-reported health declined when the sleep duration exceeded 9 h [30]. A meta-analysis revealed that the lowest risk was associated with 7 h of sleep, with all-cause mortality and CVD probabilities increasing for sleep durations exceeding 7 h or falling below this threshold [5]. Another study revealed that the point at which the U-shaped curve appears varies depending on the sleep quality of the participants: individuals with worse sleep quality tend to reach the inflection point sooner than those with better sleep quality [16]. As indicated by a joint statement of the American Academy of Sleep Medicine and Sleep Research Society [33], the optimal sleep duration varies among different populations. Below the optimal sleep duration, shorter sleep durations were associated with worse self-reported health status. This U-shaped relationship is further supported by a recent meta-analysis of 26 studies, which found that both short (≤8 h) and long (>8 h) sleep durations, as well as poor sleep quality and insomnia, were significantly associated with worse self-rated health [17].

In our study, the proportion of participants who reported a poor health status was 8.6%, which was lower than that reported in similar studies. A study conducted in Australia involving a primarily Western population indicated an 11.4% rate of poor self-reported health [30], while another study from South Korea involving an Asian population showed a 19.7% rate of poor self-reported health [32]. One possible explanation for our lower rates could be that our participants were volunteers in community screenings, which may not be fully representative of generalized health surveys. Previous research established a modest correlation between self-reported health status and physician-assessed health conditions [34,35,36]. However, some studies have identified discrepancies between self-rated health statuses and medical assessments. Inconsistent findings have emerged across studies exploring the relationship between self-reported health and smoking, alcohol consumption, and dietary behaviors [37,38]. Moreover, in Latino immigrant and African American populations in the U.S., worse self-reported health statuses were not predictive of higher BMI values [39,40]. Consequently, when formulating health policies based on self-reported health statuses, the heterogeneous characteristics of different population groups must be considered.

This is the first cross-sectional study to assess the association between sleep quality and self-reported health status in middle-aged and older Taiwanese individuals. However, our study has some limitations that should be addressed. First, the cross-sectional nature of this study limits its ability to elucidate the mechanisms and causal links between sleep quality and self-reported health status. Second, the data derived solely from community-based participants in Northern Taiwan may raise questions regarding the ecological validity of the findings and potentially introduce healthy volunteer bias. Consequently, extrapolating these results to other regions in Taiwan may be inappropriate. This potential bias toward healthier volunteers could have resulted in an underestimation of the prevalence of poor self-reported health status and may have affected our ability to adequately control for confounding factors. Third, nearly 70% of the participants were female. This imbalance might limit the generalizability of our findings to other groups, as women tend to experience more sleep disturbances than do men [41]. Fourth, data collection was conducted in 2017, based on the availability of funding and research infrastructure at that time. Nonetheless, shifts over the past 8 years related to globalization, social dynamics, and health awareness may have influenced the applicability of the results to the current context. Fifth, sleep duration, as reported in the PSQI questionnaire, showed a moderate correlation with the sleep duration objectively measured via actigraphy [42]. However, further investigation revealed that this correlation was notably strong only in the younger age group (18–32 years), whereas it was less robust in the older population (59–75 years) [43]. Future research should consider concurrently measuring both subjective and objective sleep durations, such as through actigraphy, to assess their impact on perceived health. Future research should consider employing a longitudinal study design to establish causal relationships better. Sixth, the participants were not stratified into narrower age groups, such as 50–64 and 65–85 years. Although this approach is consistent with previous studies examining sleep and health in adult populations, it may have overlooked important age-related differences. Physiological changes, including variations in hormone regulation, sleep architecture, and circadian rhythms, influence sleep quality and self-rated health. Future studies should consider age-specific subgroup analyses in order to better understand these differences. Finally, self-reported health status might be affected not only by the chronic medical conditions we have mentioned (DM, HTN, and hyperlipidemia) but also by diseases such as neurological diseases, rheumatologic diseases, cancer, or other metabolic disorders, which could confound the results. Future studies should include more participants with multiple chronic conditions.

## 5. Conclusions

In conclusion, poor sleep quality and short sleep duration were independent risk factors for poor self-reported health status, especially among community-dwelling middle-aged and older adults in Taiwan. Promoting sleep quality is key to averting the risk of potential diseases and other health-related challenges.

## Figures and Tables

**Table 1:** General characteristics of the study population according to self-reported health status.

Variables	Total (n = 850)			Self-Reported Health Status									*p* Value
				Good (n = 278)			Fair (n = 499)			Poor (n = 73)			
Age (year)	65.03	±	8.07	64.72	±	8.22	64.99	±	7.93	66.52	±	8.36	0.23
BMI (kg/m^2^)	24.51	±	3.52	24.19	±	3.01	24.57	±	3.66	25.28	±	4.20	0.05
Gender													0.06
Men, n (%)	243		28.59%	94		33.81%	129		25.85%	20		27.40%	
Women, n (%)	607		71.41%	184		66.19%	370		74.15%	53		72.60%	
Marital status													0.42
Currently in a relationship, n (%)	668		78.59%	215		77.34%	399		79.96%	54		73.97%	
Currently single, n (%)	182		21.41%	63		22.66%	100		20.04%	19		26.03%	
Education level													<0.001
Elementary school or lower, n (%)	396		46.59%	109		39.21%	248		49.70%	39		53.42%	
Middle school graduate, n (%)	172		20.24%	59		21.22%	89		17.84%	24		32.88%	
High school graduate, n (%)	199		23.41%	75		26.98%	116		23.25%	8		10.96%	
College or higher, n (%)	83		9.76%	35		12.59%	46		9.22%	2		2.74%	
Income													0.06
<20,000, n (%)	645		75.88%	197		70.86%	386		77.35%	62		84.93%	
20,000–40,000, n (%)	135		15.88%	51		18.35%	78		15.63%	6		8.22%	
>40,000, n (%)	70		8.24%	30		10.79%	35		7.01%	5		6.85%	
CES-D score													<0.001
<8, n (%)	742		87.29%	260		93.53%	439		87.98%	43		58.90%	
≧8, n (%)	108		12.71%	18		6.47%	60		12.02%	30		41.10%	
Employed													0.003
No, n (%)	662		77.88%	199		71.58%	399		79.96%	64		87.67%	
Yes, n (%)	188		22.12%	79		28.42%	100		20.04%	9		12.33%	
Exercise, n (%)	729		85.67%	254		91.37%	416		83.87%	59		80.82%	0.004
Smoking, n (%)	36		4.24%	14		5.04%	18		3.61%	4		5.48%	0.55
Alcohol consumption, n (%)	25		2.94%	7		2.52%	16		3.21%	2		2.74%	0.86
HTN, n (%)	275		32.25%	74		26.62%	164		32.87%	37		50.68%	<0.001
DM, n (%)	122		14.35%	28		10.07%	75		15.03%	19		26.03%	0.002
Hyperlipidemia, n (%)	116		13.65%	27		9.71%	68		13.63%	21		28.77%	<0.001

**Notes**: Clinical characteristics are expressed as mean ± SD values for continuous variables and as n (%) for categorical variables. *p* values were derived from the one-way ANOVA test for continuous variables and from the chi-square test for categorical variables. **Abbreviations**: BMI, body mass index; CES-D, Center for Epidemiologic Studies Depression Scale; HTN, hypertension; DM, diabetes mellitus.

**Table 2:** Sleep components according to self-reported health status.

Sleep Variable	Self-Reported Health Status						*p* Value	*p* Trend
	Good (n = 278)		Fair (n = 499)		Poor (n = 73)			
Poor subjective sleep quality							<0.001	<0.001
Total score ≦ 5	163	58.63%	221	44.29%	14	19.18%		
Total score > 5	115	41.37%	278	55.71%	59	80.82%		
Sleep quality							<0.001	<0.001
0	73	26.26%	78	15.63%	6	8.22%		
1	157	56.47%	278	55.71%	27	36.99%		
2	36	12.59%	124	24.85%	28	38.36%		
3	12	4.32%	19	3.81%	12	16.44%		
Sleep latency							<0.001	<0.001
0	132	47.48%	150	30.06%	13	17.81%		
1	67	24.10%	159	31.86%	17	23.29%		
2	54	19.42%	110	22.04%	23	31.51%		
3	25	8.99%	80	16.03%	20	27.40%		
Sleep duration							<0.001	0.004
0	49	17.63%	84	16.83%	9	12.33%		
1	74	26.62%	124	24.85%	14	19.18%		
2	127	45.68%	220	44.09%	26	35.62%		
3	28	10.07%	71	14.23%	24	32.88%		
Sleep effciency							<0.001	<0.001
0	199	71.58%	319	63.93%	37	50.68%		
1	42	15.11%	78	15.63%	8	10.96%		
2	20	7.19%	36	7.21%	7	9.59%		
3	17	6.12%	66	13.23%	21	28.77%		
Sleep disturbances							<0.001	<0.001
0	21	7.55%	25	5.01%	1	1.37%		
1	230	82.73%	392	78.56%	45	61.64%		
2	27	9.71%	82	16.43%	26	35.62%		
3	0	0.00%	0	0.00%	1	1.37%		
Sleep medication use							<0.001	<0.001
0	252	90.65%	401	80.36%	48	65.75%		
1	9	3.24%	23	4.61%	5	6.85%		
2	4	1.44%	21	4.21%	2	2.74%		
3	13	4.68%	54	10.82%	18	24.66%		
Daytime dysfunction							<0.001	<0.001
0	227	81.65%	361	72.34%	33	45.21%		
1	36	12.95%	96	19.24%	21	28.77%		
2	12	4.32%	30	6.01%	14	19.18%		
3	3	1.08%	12	2.40%	5	6.85%		

**Notes**: Clinical characteristics are expressed as n (%) for categorical variables. *p* values were derived from the chi-square test and *p* trends were derived from the Cochran–Armitage trend test.

**Table 3:** Association between sleep components and self-reported health status (poor health status as the dependent variable) by multiple logistic regression analysis.

Sleep Variable	Crude Model			Model 1			Model 2			Model 3			Model 4		
	OR	95% CI	*p* Value	OR	95% CI	*p* Value	OR	95% CI	*p* Value	OR	95% CI	*p* Value	OR	95% CI	*p* Value
Poor subjective sleep quality (PSQI > 5 vs. ≦5)	4.12	2.26–7.50	<0.001	4.08	2.24–7.45	<0.001	3.83	2.09–7.03	<0.001	3.47	1.88–6.43	<0.001	2.31	1.20–4.45	0.01
PSQI score	1.23	1.16–1.31	<0.001	1.24	1.16–1.32	<0.001	1.22	1.14–1.30	<0.001	1.21	1.14–1.29	<0.001	1.14	1.07–1.23	<0.001
Sleep quality	2.36	1.74–3.20	<0.001	2.41	1.78–3.28	<0.001	2.27	1.66–3.09	<0.001	2.18	1.58–3.00	<0.001	1.63	1.15–2.32	0.01
Sleep latency	1.63	1.30–2.04	<0.001	1.63	1.30–2.04	<0.001	1.57	1.24–1.97	<0.001	1.53	1.21–1.94	<0.001	1.30	1.00–1.68	0.049
Sleep duration	1.56	1.18–2.06	0.002	1.54	1.16–2.03	0.003	1.50	1.14–1.97	0.004	1.47	1.11–1.94	0.01	1.27	0.95–1.70	0.11
Sleep efficient	1.50	1.24–1.83	<0.001	1.50	1.23–1.82	<0.001	1.44	1.18–1.76	<0.001	1.43	1.16–1.75	0.001	1.23	0.98–1.53	0.07
Sleep disturbance	3.49	2.16–5.65	<0.001	3.59	2.21–5.85	<0.001	3.49	2.12–5.75	<0.001	3.19	1.91–5.31	<0.001	2.63	1.51–4.56	0.001
Sleep medication use	1.51	1.24–1.83	<0.001	1.48	1.22–1.81	<0.001	1.42	1.15–1.74	0.001	1.43	1.15–1.77	0.001	1.23	0.97–1.54	0.08
Daytime dysfunction	2.12	1.64–2.74	<0.001	2.14	1.65–2.77	<0.001	2.20	1.69–2.88	<0.001	2.20	1.67–2.90	<0.001	1.75	1.30–2.37	<0.001

**Notes:** Model 1: Multiple logistic regression adjusted for age and gender. Model 2: Multiple logistic regression adjusted for factors in model 1 plus marital status, education level, exercise, smoking, alcohol consumption. Model 3: Multiple logistic regression adjusted for factors in model 2 plus HTN, DM and hyperlipidemia. Model 4: Multiple logistic regression adjusted for factors in model 3 plus income, CES-D and employed. **Abbreviations:** OR, odds ratio; CI, confidence interval; PSQI, Pittsburgh Sleep Quality Index; HTN, hypertension; DM, diabetes mellitus; CES-D, Center for Epidemiologic Studies Depression Scale.

## Data Availability

The original contributions of this study are included in this article. Further inquiries can be directed to the corresponding authors.
